# Biophysical evaluation of treating adipose tissue-derived stem cells using non-thermal atmospheric pressure plasma

**DOI:** 10.1038/s41598-022-14763-0

**Published:** 2022-07-01

**Authors:** Elham shojaei, Sona Zare, Afshan Shirkavand, Esmaeil Eslami, Sara Fathollah, Parvin Mansouri

**Affiliations:** 1 School of Physics, Iran University of Science and Technology, Tehran, Iran; 2 Skin and Stem Cell Research Center, Tehran University of Medical Sciences, Tehran, Iran; 3 Laser Application in Medical Sciences Research Center, Shahid Beheshti University of Medical Sciences, Tehran, Iran; 4 Medical Lasers Research Group, Medical Laser Research Center (MLRC), Yara Institute, ACECR, Tehran, Iran; 5 Département Des Sciences Appliquées, Université du Québec À Chicoutimi (UQAC), Saguenay, QC G7H 2B1 Canada; 6 Faculty of Physics and Energy Engineering, Amirkabir University of Technology, P. O. Box, Tehran, 15875-4413 Iran

**Keywords:** Biophysics, Optics and photonics, Physics

## Abstract

Non-thermal atmospheric pressure plasma (NTAPP) is a partially ionized gas containing fast electrons and relatively slow ions. This study aims to investigate the influences of NTAPP on human adipose tissue-derived stem cells (ADSCs) and examine the feasibility of using optical spectroscopy as a non-destructive method for cell analysis. A plasma jet is used as the source of low-temperature plasma in which pure helium gas is ionized by a high voltage (8 kV) and frequency (6 kHz). ADSCs were exposed to the NTAPP for 30 s, 60 s, 90 s, and 120 s. The efficiency of the plasma treatment was investigated using flow cytometry and optical spectroscopy methods. This study compared surface markers of NTAPP treated and untreated ADSCs using CD90 and CD105 as positive markers. The result proved that NTAPP-exposed ADSCs maintain their stemming. Measuring ADSCS apoptosis by labeling Annexin V-Propidium Iodide showed that the plasma at short exposure time is relatively non-toxic. However, a longer exposure time can lead to apoptosis and necrosis. Moreover, Cell cycle analysis revealed that NTAPP accelerates the cell cycle in very low doses and can cause proliferation. In this experiment, flow cytometry measurements have been used to determine oxidative stress. The results showed that with increasing plasma dose, intracellular ROS levels reduced. This data also suggests that intracellular ROS are not responsible for the cells' viability. Furthermore, we used reflectance spectroscopy as a non-destructive method for evaluating treatment response and comparing this method with cell analysis techniques. The results indicate spectroscopy's efficiency as a method of cell analysis. This study suggests that NTAPP would be an efficient tool to improve ADSCs culture's efficiency in vitro; thus, we support the potential applications of NTAPP in the field of stem cell therapy and regenerative medicine.

## Introduction

Non-thermal atmospheric pressure plasma (NTAPP) is a partially ionized gas at atmospheric pressure^1,2^. Several studies have tried to take advantage of NTAPP for biomedical applications due to the controllability of the chemistry and kinetics of plasma^3–8^. Plasma generates controllable amounts of short-lived reactive oxygen and nitrogen species (ROS and RNS, respectively). This Reactive species can be altered by adjusting frequency, voltage, and feeding gases. RNS and ROS act as primary cell signaling molecules to regulate cellular and physiological functions inside the human body. External administration of RNS or ROS by NTAPP can boost the natural physiological processes^9–14^.

In these experiments, we have used human adipose tissue-derived stem cells (ADSCs). ADSCs can be isolated from adipose tissues by liposuction, and they can be used in stem cell therapy and regenerative medicine. However, in general, the cultivation of ADSCs is difficult. Only a minimal number of ADSCs can be obtained from the tissues of a patient, and they have inefficient proliferation. Also, stem cells' characteristics are challenging to maintain during culture in vitro, and they easily undergo rapid senescence^15–17^. ADSCs are mesenchymal stem cells with the capacity for self-renewal and the ability to differentiate into various lineages. Multipotency allows these cells to differentiate into specific cell types such as osteoblasts, adipocytes, neurons, and chondrocytes^15,18^. These properties make MSCs an appropriate resource for cell-based therapy for treating diabetes mellitus, cardiovascular disease, leukemia, neurodegenerative diseases, and cartilage disorders^19^, as well as applications in reconstructive or tissue engineering medicine^20^. MSCs exist in almost all tissues, including bone marrow, blood vessels, skin, brain, and muscle^21^. However, Adult tissues have few stem cells, and ex vivo isolation and maintenance of stem cells is difficult. On the other hand, Adipose tissue provides for a large volume of tissue extraction with minimal morbidity. Furthermore, ADSCs can tolerate freezing and thawing procedures without losing their multipotential characteristics. They also exhibit a normal diploid karyotype and, as we said, can differentiate into a variety of MSCs, including adipocytes, even after extensive expansion at the single-cell level^22^. Therefore, ADSCs can be used in stem cell therapy and regenerative medicine as an accessible adult stem cell source^18,23^. ADSCs, interestingly, respond to atrial natriuretic peptides (ANP)^22^. Moreover, compared to other cell lines of preadipocytes, differentiated ADSCs can secrete adiponectin and leptin at levels similar to those found in isolated human adipocytes^24^. This means ADSCs can become a new and beneficial tool in pharmacological research. After transplantation into animal models, ADSCs can participate in muscle regeneration^25^ and promote neovascularization^26,27^. This can highlight the idea that adipose tissue can be used as a new source of stem cells and has therapeutic potential.

This study aimed to investigate plasma interaction with ADSCs to find other potential applications, including wound healing improvement or the extreme precision removal of pathological tissues or cells while causing minimal injury to the body. We have also evaluated the possibility of using optical spectroscopy as a non-destructive method for cell analysis. ADSCs proliferation and death following plasma treatment were measured by flow cytometry method and optical spectroscopy approach. The effect of NTAPP on the ADSCs cell cycle and intracellular ROS was investigated, and finally, mechanisms of NTAPP effects were explored. We showed that while high dose NTAPP induces ADSCs death, lower doses enhance proliferation without affecting their stem cell characteristics. Our findings also showed that reflectance spectroscopy could provide quantitative data for evaluating treatment response that corresponds with cell biology techniques. Taken together, we support the potential application of NTAPP in regenerative medicine and stem cell therapy. The findings and conclusions that have been taken from this study reinforce our knowledge of NTAPP interactions with stem cells and will be beneficial in the future development of NTAPP technology.

## Material and methods

### Cell preparation, culturing, and isolation of human ADSCs

Adipose tissue was obtained from patients referred to the Skin and Stem Cell Research Center (SSRC), Tehran University of Medical Sciences, for liposuction. Written informed consent was obtained from patients to use their discarded tissue to isolate mesenchymal stem cells. All methods were carried out following relevant guidelines and regulations, and also SSRC approved all experimental protocols. The research was approved by the ethics committee of Shahid Beheshti University of Medical Sciences, IR.SBMU.RETECH.REC.1400.936.

ADSCs were isolated from the tissue and then repeatedly washed with Phosphate-buffered saline (PBS). Next, 0.0075 percent collagenase (Sigma-Aldrich, MO, USA) was added to the tissue sample at 37 °C, and then the samples were shaken for an hour. In order to remove the top layer of fat, oil, and The Collagenase solution layer, the sample was centrifuged at 1000 rpm for ten minutes, and the stromal vADSCsular fraction (SVF) pellet was obtained. The pelleted SVF was suspended for 10 min at room temperature in 155 mM NH4Cl for lysing of red blood cells. Then using centrifugation at 1000 rpm for 10 min, ADSCs were collected.

ADSCs were holding in Dulbecco's modified Eagle's medium (DMEM), Ham's F-12 supplemented with 10% (v/v) fetal bovine serum (FBS; Sigma-Aldrich, MO, USA), and 10 ml/l penicillin–streptomycin (GIBCO, NY, USA). ADSCs were maintained and grown in DMEM containing 10% FBS and 10 ml/l penicillin–streptomycin. All cells were maintained at 37 °C in an atmosphere containing 5% CO2.

For Cells Passaging, ADSCs were trypsinized and refreshed weekly, and only cells from 4 or fewer passages were used in the experiments.

### Plasma generation and experimental setup

**Figure 1:**
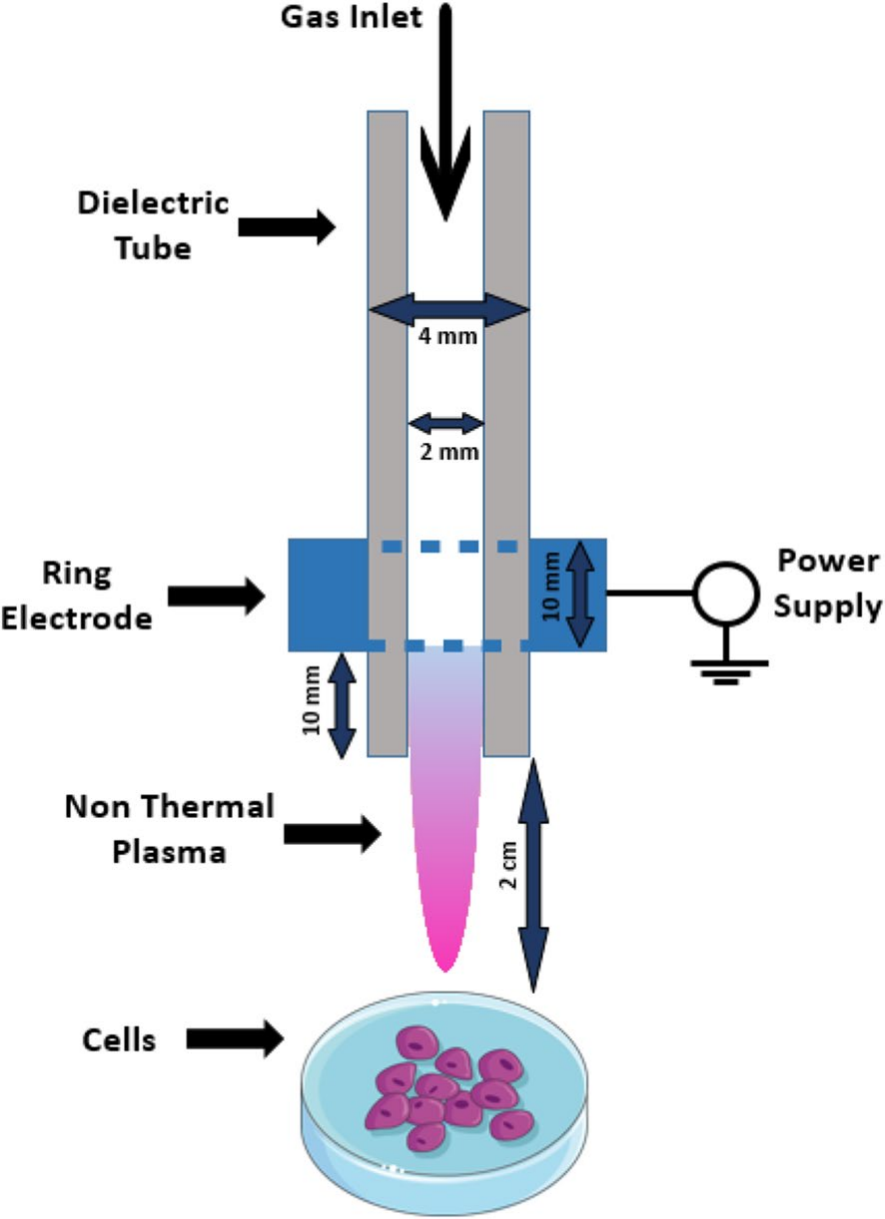
Helium-based plasma jet device used for NTAPP generation, Schematic of Inner components of the device.

The plasma source used in this study was the plasma jet previously used in wound healing in diabetic rats^28^ and treating diabetic foot ulcers^29^. The plasma jet used in this study consists of an insulating Pyrex tube (ID: 2 mm and OD: 4 mm) as a tube to control the discharge of working gas. A copper wire with 10 mm width was wrapped around the tube to work as a power electrode. The distance between the powered electrode and the nozzle tip was 10 mm. The virtual ground electrode of the space has been used in this configuration. Plasma was generated by applying a pulsed DC high voltage between the power electrode and the sample using a high voltage power supply. The repetition frequency, applied voltage, and duty ratio were 6 kHz, 8 kV, and 15%, respectively. A gas flow of 2 lit/min for 99.999% pure helium (He) was used. The distance between the nozzle tip and cells was 2 cm. The schematic of the experimental setup is shown in Fig. 1.

### Plasma treatment

Human ADSCs were isolated from adipose tissue and characterized as plastic adherent cells with fibroblastic morphology. After four successive passages, we obtained a homogeneous ADSCs population. Before treatment, 3 × ${10}^{4}$ cells seeded in multiple well plates (6 well plates) were incubated for 24 h at 37 °C in a humidified atmosphere with 5% CO2. Cells were exposed to the NTAPP for the 30 s, 60 s, 90 s, and 120 s. 0 s treatment cells were considered as the control group. The cells were incubated for 48 h after treatment. The distance between the cells and the device was fixed to 2 cm, and 1.5 ml of medium was used. Flow cytometry Analysis (Evaluation of ADSCs surface markers and Immunophenotype, Analysis of apoptosis, cell cycle analysis, and Detection of intracellular ROS) and Optical reflectance spectroscopy have been done 48 h after treatment.

### Evaluation of cell morphology after treatment

We examined and imaged morphological changes of the cells after plasma treatment using an IX70 inverted phase-contrast light microscope (Olympus, Japan).

### Evaluation of ADSCs surface markers and Immunophenotype

To detect specific stem cells' surface markers, at 48 h after exposure to NTAPP, ADSCs were trypsinized with 0.25% trypsin–EDTA (GIBCO, NY, USA), washed, and centrifuged for 5 min at 1500 rpm. Cells were incubated with anti-CD45-fluorescein isothiocyanate (FITC; eBioscience, CA, USA), anti-CD105-phycoerythrin (PE; eBioscience, CA, USA), conjugated anti-CD90 (eBioscience, CA, USA), and appropriate isotype-matched antibodies for 30 min in the dark. 10,000 cells per assay were counted using a FACSCalibur flow cytometer (BD Bioscience, CA, USA).

### Analysis of apoptosis

ADSCS apoptosis was measured through the labeling of Annexin V-Propidium Iodide. This test is based on the protein Annexin V's ability to bind to phosphatidylserine (PS), which is translocated from the Interior cell membrane leaflet of viable cells to the outer in apoptotic cells (PS appears on the surface of necrotic cells as well). Propidium Iodide (PI) is a DNA-binding fluorescent reagent that penetrates damaged cell membranes. Adding PI allowed the distinguishing of viable cells (AnnVneg/PIneg), early apoptotic cells (AnnVpoz/PIneg), late apoptotic cells (AnnVpoz/PIpoz), and necrotic cells (AnnVneg/ PIpoz)^30,31^.

The apoptosis assay was performed using Annexin V/ Pi method, following the manufacturer's instructions (Annexin V-FITC kit, Bender MedSystems, Austria). After treatment, the harvested cells were washed in PBS, resuspended in 100 μl of binding buffer, and stained with 5 μl FITC-conjugated Annexin-V for 15 min in the dark at room temperature. The samples were washed and resuspended in 250 μl binding buffer and then incubated with 5 μl Propidium Iodide (PI; Sigma-Aldrich, MO, USA) for 10 min. The results were analyzed on the BD FACSCalibur cytometer.

### Cell cycle analysis

After treatment, the harvested cells were detached for the cell cycle analysis with 0.25% trypsin- EDTA (GIBCO, NY, USA), washed with PBS and then fixed with ice-cold 70% ethanol for 2 h. Cells were rewashed with PBS and treated with 50 μg/ml RNase A (Bio Basic Inc., ON, Canada) for 30 min. After that, cells were incubated with Propidium Iodide (PI; Sigma-Aldrich, MO, USA). Flow cytometry analysis was performed using the BD FACSCalibur cytometer.

### Detection of intracellular ROS

2, 7 -Dichlorofluorescin diacetate (DCFH-DA, Sigma), a stable non-fluorescent compound, was produced from dissolving 0.02 mmol of DCFH-DA in 1 mL of DMSO. DCFHDA underwent deacetylation by intracellular esterases when crossing the membrane and converted to DCFH. In the presence of active radicals within the cell, the non-fluorescent DCFH can oxidize and produce the highly fluorescent form DCF22. The cells were harvested and washed within PBS and incubated with 50 μmol/L 2, 7 -Dichlorofluorescin diacetate (DCFH-DA, Sigma) at 37 °C for 2 h. Then the cells were washed and placed on ice in the dark. Before reading, 5 μl of Propidium Iodide (PI; Sigma-Aldrich, MO, USA) was added to the samples. The analysis was performed with the FACS Calibur FCM. The labeled cells were excited at 488 nm, and the emissions were detected at 520 nm and 630 nm.

### Optical reflectance spectroscopy

We have used an optical reflectance spectroscopy system, a diffusion reflected one, as a non-destructive method for some physical analysis. Illumination for the spectra measurements was 400–1000 nm wavelength range. A miniature CCD-based fiber optic spectrometer (USB 2000; Ocean Optics) was applied (Fig. 7a). The spectroscopy experiments for recording reflectance spectra were conducted on the cell samples between the control group and the cell samples between 30, 60, 90, and 120 s of plasma treatment. To ensure more reliable cell spectroscopic signals and minimize bias in recording spectra, we first performed background subtraction of the plate and media (with or without soluble drug) as we had the option in the spectroscopy system^32–34^.

### Statistical and data analysis

Statistical analysis of data was performed using SPSS Statistics 21.0. One-way analysis of variance (ANOVA) followed by Tukey's post-hoc test was performed to determine statistical significance. Data were considered statistically significant for *P* < 0.05 (marked as a single star), *P* < 0.01 (double star) and, *P* < 0.001 (triple star) were considered as extremely significant compared with the control. Flow cytometry data were acquired and analyzed using a FACSCalibur flow cytometer (BD Bioscience, CA, USA) as a prepaid service in out lab.

## Results

### Observation of morphological changes by microscopy

**Figure 2:**
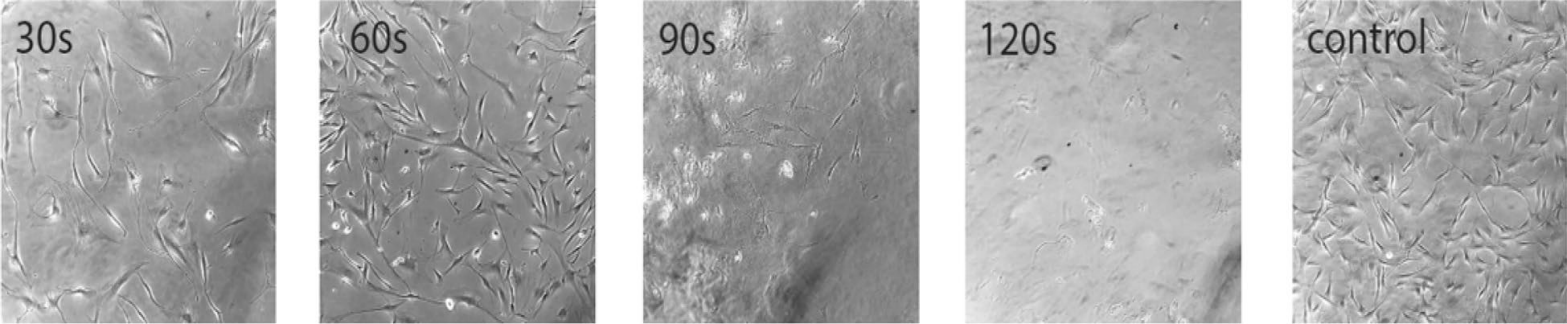
Morphological changes in ADSCs after treatment with NTAPP for the 30 s, 60 s, 90 s, and 120 s.

Normal ACSCs have a polyhedral shape and are adherent. However, dying cells shrunk, rounded and detached themselves from the plate. Our first observations indicate that with increasing plasma dose, the number of dying cells went up (Fig. 2). Our result shows that more and more cells shrunk, rounded, detached themselves from the plate, and finally died with increasing plasma dose.

### NTAPP-exposed ADSCs keep their stemming

**Figure 3:**
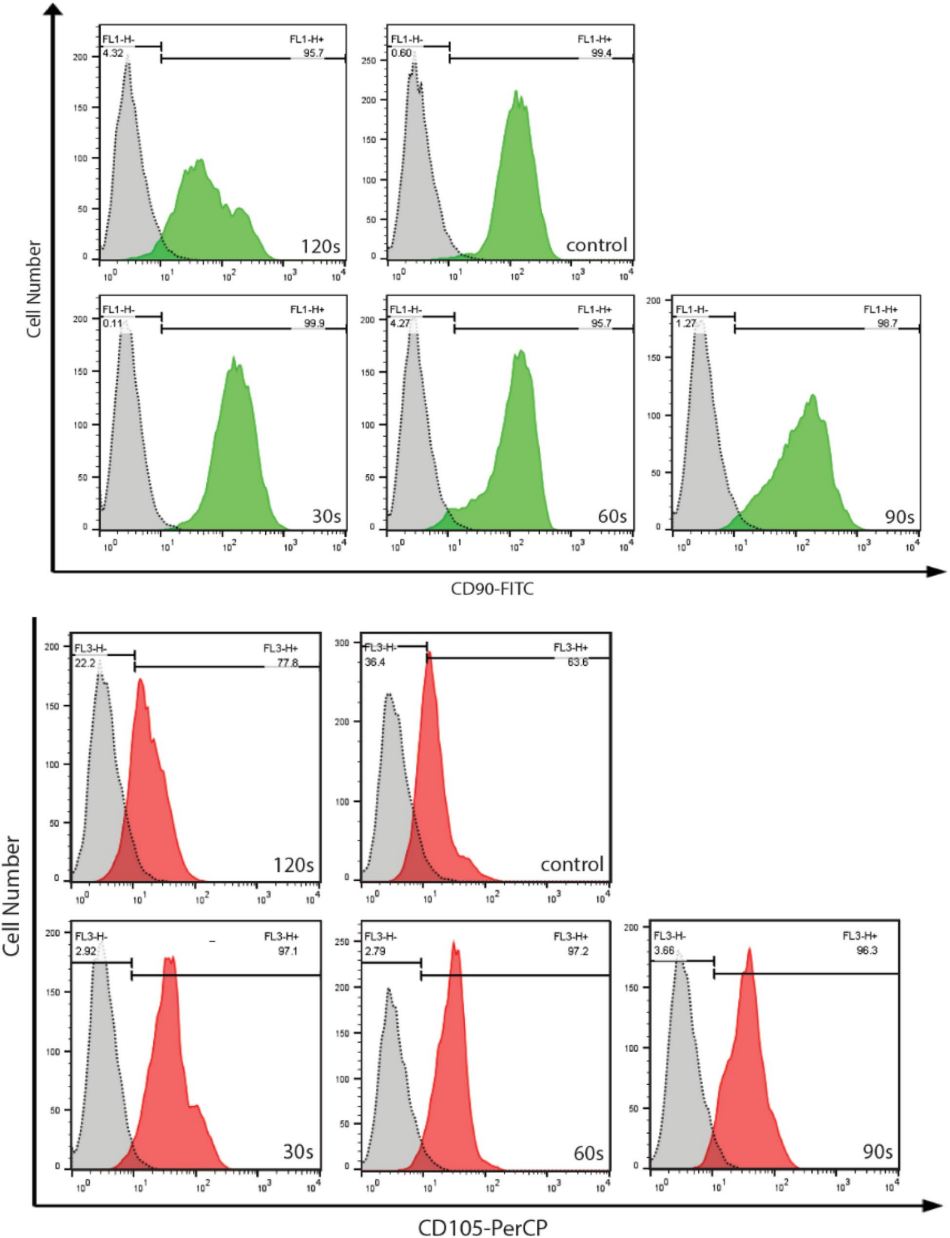
NTAPP exposed ADSCs maintain their stem cell properties. CD90 and CD105 were used as positive markers for the analysis of ADSCs.

To use NTAPP to enhance the proliferation of ADSCs for various applications, after exposure to NTAPP, the stem cell properties of ADSCs should be maintained. Characteristics of stemness of NTAPP treated and untreated ADSCs were compared using CD90 and CD105 as positive markers. The result demonstrated that both treated and untreated ADSCs showed high CD90 and CD105 expression (Fig. 3). These findings confirm that NTAPP treatment does not alter the characteristics of stemness of ADSCs.

### Adipose tissue-derived stem cell death in response to non-thermal plasma

Staining of Annexin V / Propidium Iodide (AnnV / PI) is an assay based on the protein Annexin V being able to attach to the PS and PI ability to bind to DNA directly^35^. We can divide the data obtained from this test into four categories.

Quadrant 4 (Live cells): Double negative as neither Annexin V nor PI staining could have been detected.

Quadrant 3 (Early apoptotic cells): Annexin is positive, and PI is negative as the membrane stays intact.

Quadrant 2 (Secondary necrotic and late apoptotic): Double-positive (membrane damage leads to pi staining penetration).

Quadrant 1 (Nuclei without plasma membrane): Necrotic cells with positive PI and negative Annexin (no membrane left for the Annexin to bind)^30,31^.

**Figure 4:**
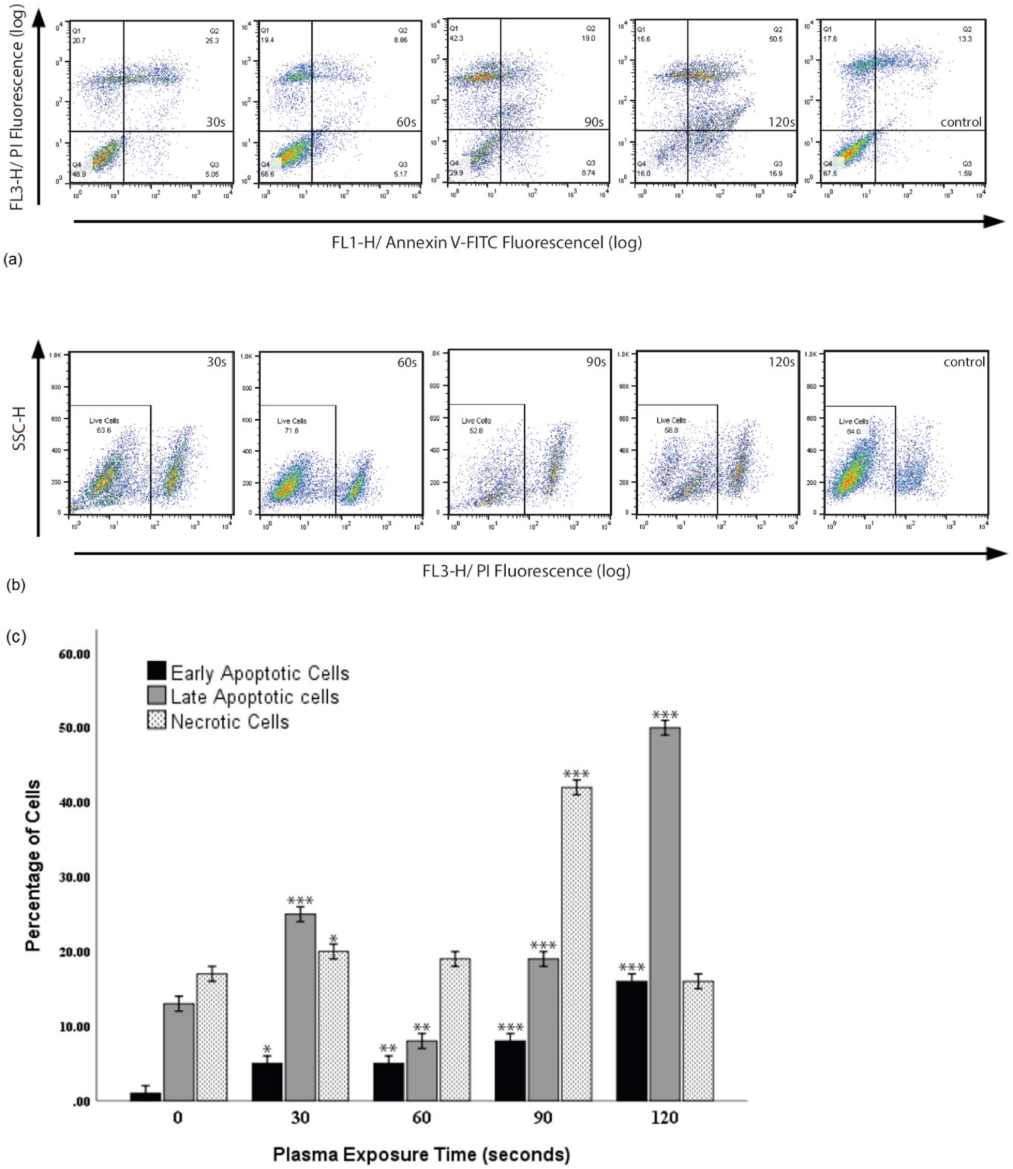

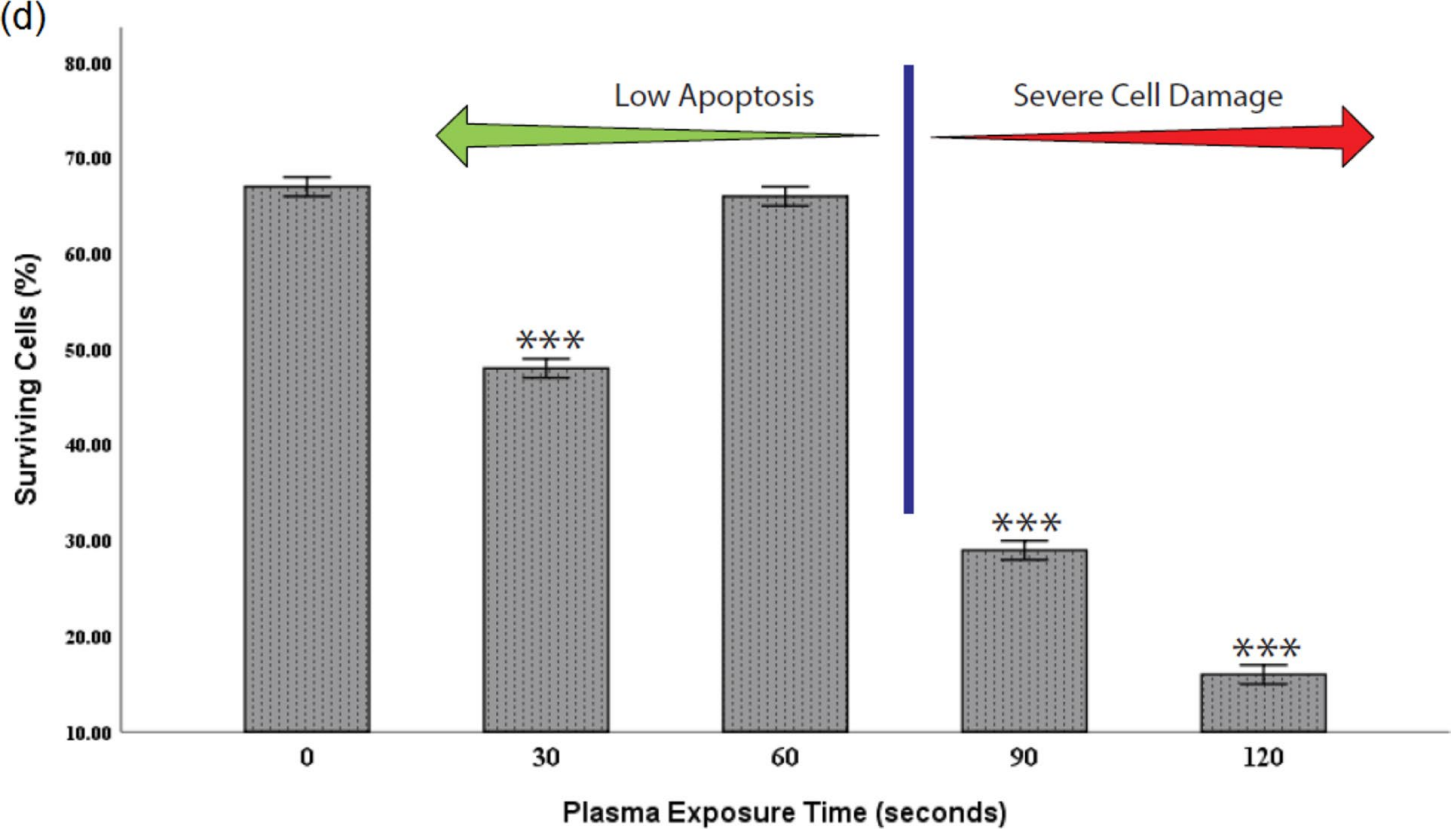
Quadrant 4 (live cells), Quadrant 3 (Early apoptotic cells), Quadrant 2 (Secondary necrotic and late apoptotic), and Quadrant 1 (Necrotic cells). (**A**,**C**,**D**) Cell viability was measured by Annexin V / Propidium Iodide assay. Data were considered statistically significant for *P* < 0.05 (marked as a single star), *P* < 0.01 (double star) and, *P* < 0.001 (triple star) were considered as extremely significant compared with the control. (**B**) Propidium Iodide assay was used to confirm AnnV/PI results.

Plasma-treated ADSCs with short exposure times up to 30 s showed a few dead cells (mostly in late apoptotic and necrotic cell stages). However, with an increase in treatment time up to 60 s, fewer cells were destroyed (mostly in the apoptotic stage), verifying that the plasma at short exposures is relatively non-toxic. On the other hand, many dead cells were evident in the 90 s (Majorly in the necrotic cell stage) and 120 s (almost all in the double-positive stage) (Fig. 4A,C,D). We used the assay of the viability of cells with Propidium Iodide to confirm AnnV/PI results (Fig. 4B). Plasma-treated ADSCs with short exposure times showed a few dead cells; however, longer exposure times induce apoptosis and necrosis.

### Distribution of the cell population during the cell cycle under treatment with NTAPP

Since the accelerated cell cycle represents an increase in proliferation, we examined how NTAPP treatment influenced cell cycle progression in ADSCs. The cell cycle consists of phase G1, phase S, phase G2, and phase M. During step S, DNA is duplicated, and cell growth occurs in phases G1 and G2^36,37^.

In the control group, the largest proportion of cells belonged to phase G1 at around 63%. The figure for phase S stood at 22.46%, while that of phase G2 was lower at nearly 5% in the 0 s treatment group. In the 30 s and 60 s treatment, more cells belonged to phase S and G2 of the cell cycle than the control group. Nevertheless, fewer cells were in phase G1 in the 30 s and 60 s treatment than in the control group. Since proliferation increase would only be possible when the cell cycle is accelerated, these results suggest that NTAPP, in very low doses, caused a dose-dependent increase in the number of cells.

**Figure 5:**
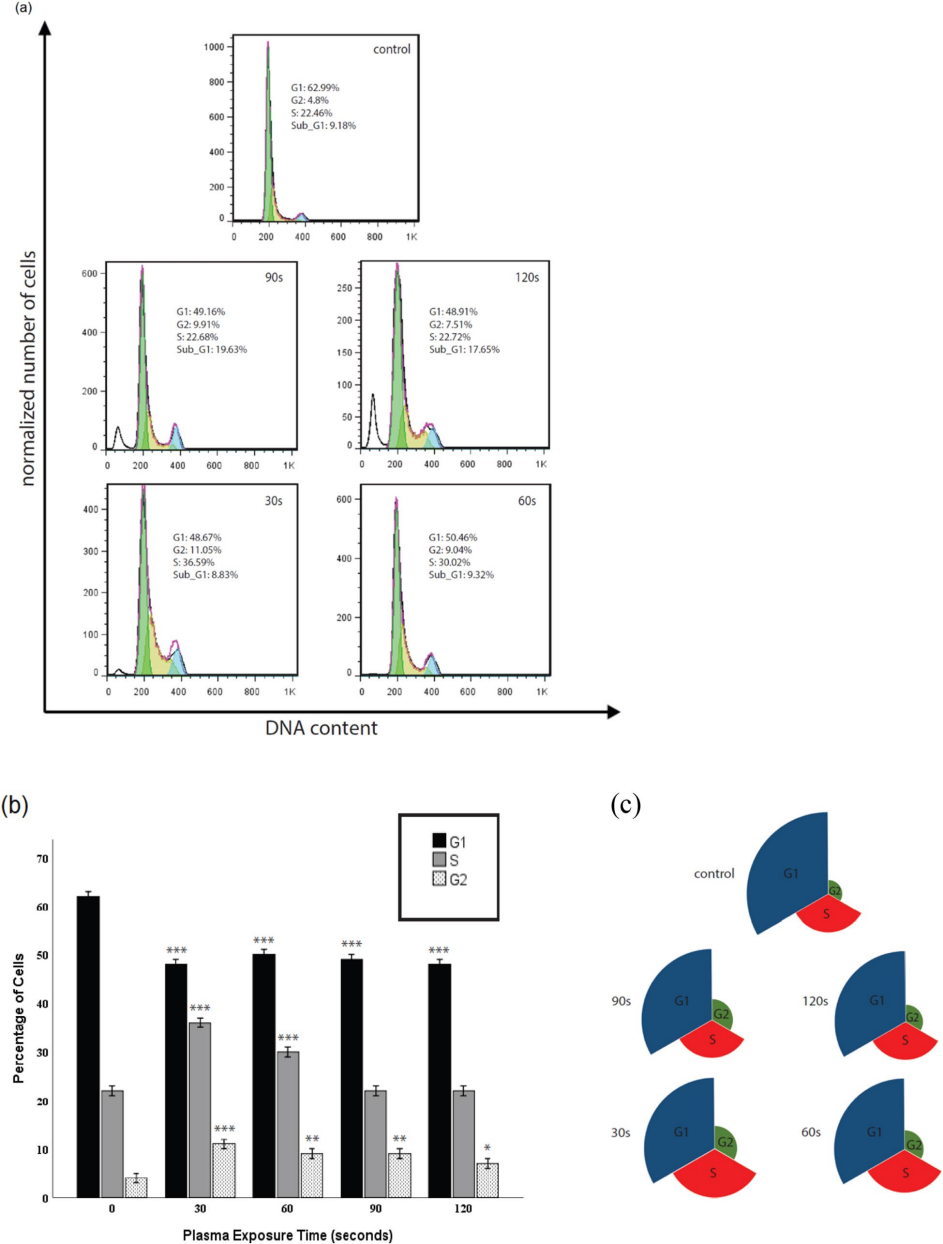
(**a**) Representative of the cell cycle of ADSCs obtained from flow cytometer. (**b**) Data were considered statistically significant for *P* < 0.05 (marked as a single star), *P* < 0.01 (double star) and, *P* < 0.001 (triple star) were considered as extremely significant compared with the control. (**c**) Circular statistics.

In the 90 s and 60 s treatment, we observed no significant difference in the proportion of cells in the phase S of the cell cycle compared to the control group (around 22%). More cells belonged to the G2 phase in these groups, whereas fewer cells existed in the G1 phase than in the control group. These results indicate a decrease in cell viability in a very high dose of plasma treatment. One of the main characteristics of apoptosis is the Fragmentation of the internucleosomal DNA. In the DNA frequency histograms, apoptotic cells can be categorized as cells with a fractional DNA content (sub-G1)^38^. In this experiment, increased cell death was observed with increasing plasma dose, and increased cell death was associated with a rise in the percentage of cells in the sub-G1 phase (Fig. 5a,b). NTAPP affects all cell cycle stages (Fig. 5c); however, the cell response is different depending on the cell cycle phase in which a cell exists.

### Determination of intracellular ROS level after NTAPP treatment

Suggesting that ROS contributes to regulating cellular functions of ADSCs, after NTAPP exposure, we evaluated the level of intracellular ROS in ADSCs. ROS continuously produced in normal cells at a restricted level. ROS level could be significantly increased in response to a wide range of pathophysiological. Extremely high ROS level will lead to biological macromolecules oxidization and cellular pathology, which finally leads to cell death^39,40^. Due to their short lifetimes, measuring ROS is highly challenging. In our study, flow cytometry measurements have been used to determine oxidative stress. The basis for this cellular assay is evaluating the fluorescence intensity^39–41^.

**Figure 6:**
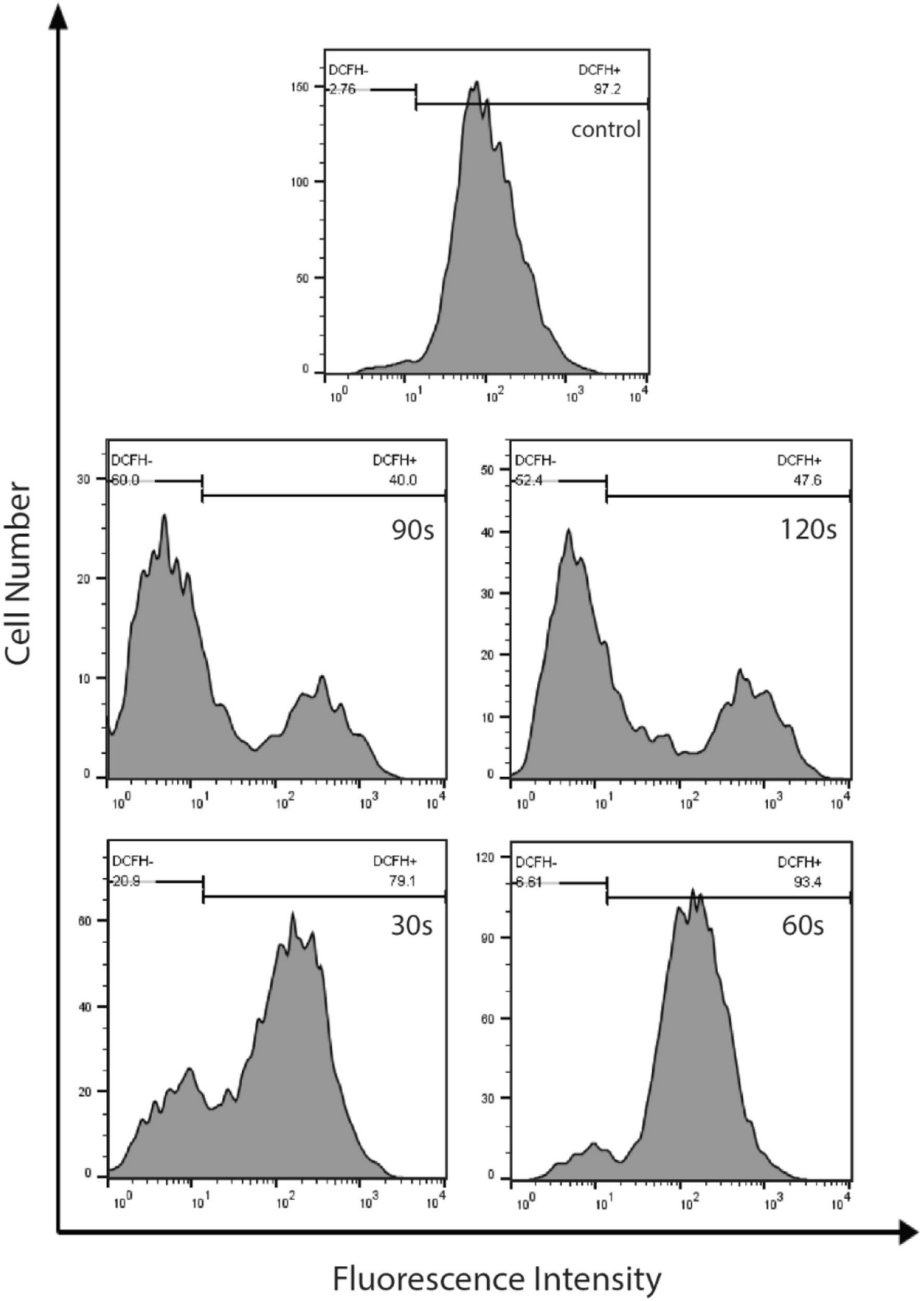
ROS are not responsible for NTAPP-induced apoptosis of ADSCs. The increase in ROS levels is indicated in the flow cytometry measurements as a shift in the histograms showing the frequency distribution of DCF fluorescence values versus the number of events or cells. In this experiment, intracellular ROS levels were reduced with increasing plasma dose.

The flow cytometry measurements indicate the increase in ROS levels as a shift in the histograms showing the frequency distribution of DCF fluorescence values versus the number of events or cells. In this experiment, intracellular ROS levels were reduced with increasing plasma dose. The ROS level was lower in the 30 s and 60 s treatment than in cells in the untreated controls. Although the figure for the 30 s was approximately equal to that of the 60 s treatment, fewer cells were destroyed in the 60 s treatment than in the 30 s treatment (Fig. 6).

### Optical reflectance spectroscopy

**Figure 7:**
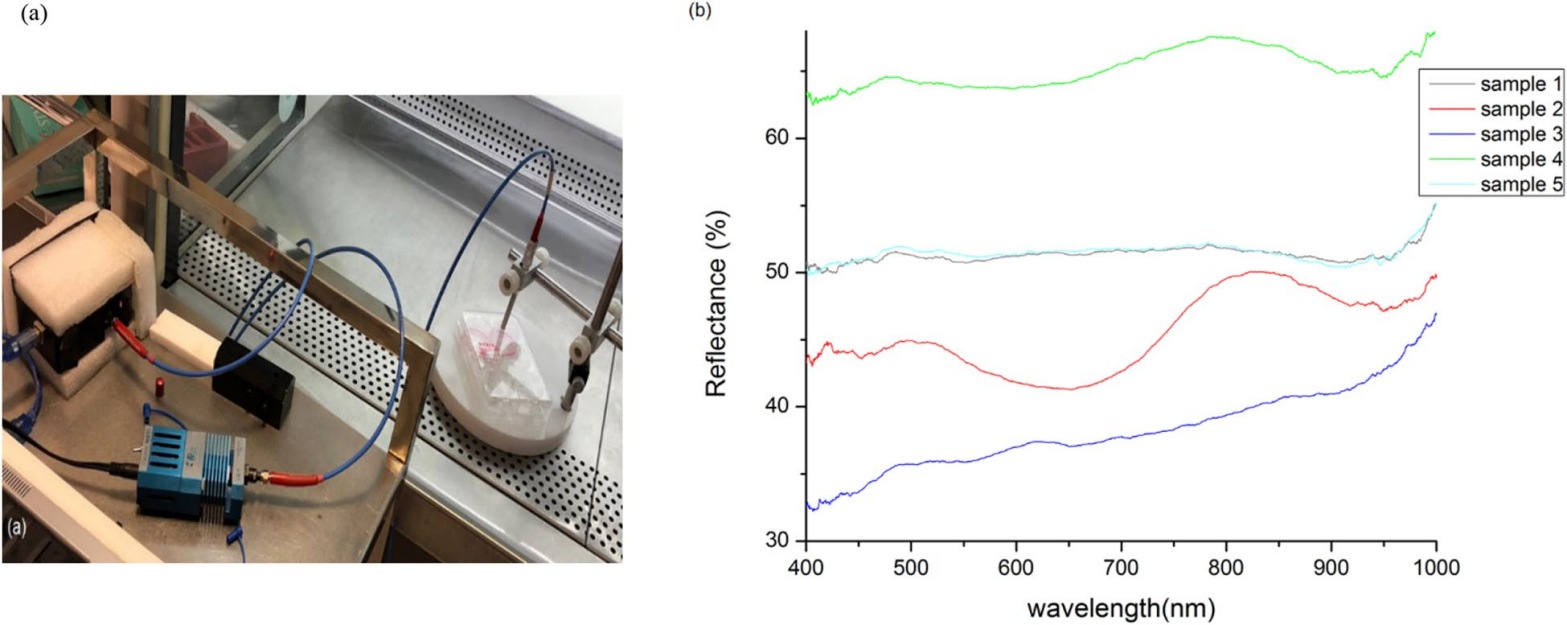
Optical reflectance spectroscopy (**a**) setup (**b**) resulted spectra. Samples 1, 2, 3, 4, and 5 are 30 s, 60 s, 90 s, 120 s, and 0 s treatment, respectively.

We used reflectance spectroscopy as a non-destructive method for evaluating treatment response and comparing this method with cell analysis techniques. There was a similarity in the refractive index of cell samples between 30 s and the control group. It is worth checking that there is a similarity in the stemness characteristics of ADSCs between the 30 s and 0 s treatment. Based on the reflectance spectra, in the 60 s treatment, the reflectance of samples decreased, and there was a peak around 800 nm, while in the control group and 60 s group, a similar trend was seen. There was a considerable decrease in the refractive index of cells in the 90 s treatment. In the group of 120 s irradiation, a very polished and reflected trend seems to be shown like a mirror, and it seems that the cellular characteristic is changing. Flow cytometry data seems to agree with the spectroscopic data, which evaluates the behavior of optical properties for control groups of 30 s, 60 s, and 90 s, showing the spectroscopy's efficiency as a method of cell analysis (Fig. 7). Reflectance spectroscopy was used as a non-destructive method for evaluating treatment response and comparing this method with cell analysis techniques. Our spectroscopic data shows the spectroscopy's efficiency as a cell analysis method.

## Discussion

Up to now, NTAPP has been researched for its clinical application^42–45^; however, several unanswered questions remain about the role of NTAPP in proliferation activation. It is worth mentioning that it is difficult to compare plasma sources thoroughly. The NTAPP working parameters are nearly impossible to standardize, and they are separately defined for every single source. This leads to a hinder in the interpretation of the plasma-induced effects. Therefore, similar experiments in many different laboratories using the same standardized source will be very useful to perform.

The ADSCs isolated for this research shared the characteristics of other postnatal MSCs derived from various sources^46^, including typical fibroblastic morphology, adherence to plastic, and MSC specific surface markers expression. This study used a helium-based plasma jet as an NTAPP device generating multiple intracellular ROS/RNS. To determine whether the NTAPP affected the viability of the ADSCs, primary cells isolated from adipose tissue were cultured and treated by plasma jet during four exposure times (30, 60, 90, and 120 s).

We demonstrated that NTAPP at short exposures is relatively non-toxic, although the number of dead cells rose as the exposure time increased. Cell cycle analysis suggests that NTAPP, in very low doses, promotes the proliferation of ADSCs while maintaining their stem cell surface markers. We also showed that, although there is a direct relationship between decreasing ROS and increasing cell death, increasing ROS level is not the only factor influencing cell death. Collectively, we strongly suggest that NTAPP can improve ADSCS culture's efficiency in vitro; thus, we support the potential applications of NTAPP in the field of stem cell therapy and regenerative medicine.

We may be able to destroy ADSCs without Considerable necrosis and subsequent inflammation by controlling plasma dose. We showed that prolonged exposure time to plasma could lead to cell death. In this study, the best time for cell proliferation was 60 s and the optimal time for cell death was 120 s, which did not cause necrosis. Our cell cycle data show that NTAPP affects all cell cycle stages; however, the cell response is different depending on the cell cycle phase in which a cell exists. Our results show that cells in the untreated group were mainly in phase G1. The short plasma exposure time led to an increase in the cell number in phase S and G2 and a decrease in cell number in phase G1 compared to the control group (No changes were observed in the phase sub G1). This suggests that NTAPP, in low doses, caused a dose-dependent increase in the number of cells and cell proliferation.

On the other hand, the long exposure time of plasma contributes to increased cell number in the G2 / M fraction and sub-G1 and decreases in cell number in phase G1. In phase S of the cell cycle, there was no significant difference in the number of cells in the 90 s and 120 s treatment compared to the control group. These results indicate increased cell death in a high dose of plasma treatment. Generally, plasma decreased the number of cells in phase G1, although the plasma effect on this phase is not dose-dependent. As we said, the control group cells were mostly in phase G1. With short-term plasma exposure, more cells were in phase S, and with a further increase in exposure time, most cells were in phase sub G1 compared to 0 s treatment.

Worth mentioning that although fewer dead cells were observed in the 60 s group than in the 30 s, the number of cells in phase S in the 30 s was higher. Generally, differentiation and proliferation are poorly compatible, and the process of differentiation is identified as a sequential event following weakened cell proliferation. In other words, when stem cells differentiate into specialized cells, intracellular signals and the growth factors responsible for cell growth are inhibited^47,48^. Therefore, we can suggest that the decrease in the number of cells in phase S in the 60 s treatment was possibly a prerequisite for their subsequent induction of differentiation. More research is required to describe these growth inhibitor properties and induce differentiation by NTAPP.

As previously noted, it is not easy to fully describe plasma itself. Besides, its biologically active components are much more complicated, so it is hard to examine their effects, and the mechanisms involved are even more challenging to explain. Our initial step is that plasma generates a rich mixture of ROS and RNS, which are most likely to play a crucial role in the phenomena mentioned to date^13,49,50^. Historically, ROS and RNS have been considered 'bad' and strongly related to free-radical aging. However, nowadays, prevailing thought focuses on the role of ROS and RNS in a wide range of biological processes involved in the repair and protection of organisms and cells^51^. Although large doses of ROS and RNS are harmful to any cell and organism, the delivery of low dosage may help treat a wide variety of indications^12,13,49–52^.

ROS could be in charge of the various biomedical effects of NTAPP^53–55^. At low concentrations, ROS can operate as a signaling molecule to modify cell processes and promote cell proliferation, differentiation, and migration^56–58^. ROS also helps stem cells maintain their stemness^59^. In contrast, higher ROS levels induce cell senescence and apoptosis, and too high concentrations of ROS cause non-specific cell death, which is probably necrosis^60–62^. Laurent et al. have indicated that low exogenous hydrogen peroxide level increased NIH 3T3 fibroblast proliferation while high concentrations of hydrogen peroxide have contributed to cell death^57^. In a study conducted by Dehui Xu (2015), it was shown that H2O2 and O2- are the main reactive species that trigger cell death. However, since each species alone was inadequate to obtain this result and cells express various iron proteins, they suggested that the OH radical produced by the Haber–Weiss reaction causes cell death. Iron proteins such as lactoferrin receptor, transferrin, and ferritin could catalyze H2O2 and O2–radicals into extremely reactive OH radicals that could seriously damage several macromolecules, such as DNA, lipids, and proteins^14^.

On the other side, RNS such as Nitric oxide is associated with different physiological functions^63^, such as the proliferation and differentiation of neural stem cells^64^. Besides, NO plays an intracellular messenger role in biological functions, including apoptosis^65–67^. A low NO level promotes cell proliferation^68^, and a high NO level contributes to cell cycle arrest and cell death^69^. In 2016, Jeongyeon Park and her team showed that helium-based NAPP increased the proliferation of ADSCs by activating ERK1/2, Akt, and their downstream NF-κ B via NO. Their result showed that the increased proliferation of ADSCs is triggered by NO rather than ROS^19,70,71^. Another study in 2015 showed that nitrogen species alone, such as electronically excited N2 and N ions, do not lead to cell death, and the production of RNS, such as NO or peroxynitrite (ONOO–) which can cause cell death, requires the presence of oxygen to be useful^12^. This emphasizes the significance of the presence of oxygen for plasma-induced cell death. In 2020, Park J et al. studied how CAP activates stem cell proliferation through epigenetic mechanisms. After analyzing the entire genome expression profiles of ASCs, they discovered that CAP upregulated genes for chemokines, cytokines, and growth factors while downregulating genes for intrinsic apoptotic pathways. They showed that NO produced from CAP was mainly responsible for plasma-induced epigenetic modifications at the mRNA and protein levels^71^.

Therefore, while some studies prove that ROS positively affects cell proliferation and some show they do not, all studies claim that ROS can cause cell death in higher doses. RNS, on the other hand, cannot cause cell death on their own, even in high doses, and the presence of oxygen is required for effective cell killing by plasma. However, RNS are the main component responsible for cell proliferation.

We also measured the intracellular ROS level in ADSCs after NTAPP exposure using DCFH-DA, a fluorogenic dye that monitored ROS activity. In our study, although cell death was different between the 30 s and 60 s of treatment, ROS level in 30 s treatment was the same as 60 s treatment. This data suggest that intracellular ROS are not responsible for the viability of cells. It is worth mentioning that intracellular ROS in a higher dose of plasma treatment decreased dose-dependent. This can be caused by a decrease in the total number of cells. Consistent with our result, other research groups have reported that the increased proliferation of ADSCs after NTAPP exposure is not triggered by intracellular ROS^70^.

We also used reflectance spectroscopy as a non-destructive way to evaluate treatment response and compare this approach to cell analysis techniques. The data for the 30 s group overlap control group results, and it is important to point out that there is an overlap between characteristics of stemness of ADSCs in the 30 s and 0 s treatment. There was a dose-dependent decrease in the refractive index of samples in the 60 s and 90 s treatment compared to the control group. The 120 s figure shows reflective and mirror behavior, and the cellular characteristic appears to be changing. These results show the spectroscopy's efficiency as a method of cell analysis. The findings of this study reinforce the ability of NTAPP to control the viability of stem cells without changing their stemness. The effect of NTAPP on other stem cells needs to be studied to improve NTAPP as a valuable method for biomedical application.
